# Rapid evaluation and quality control of next generation sequencing data with FaQCs

**DOI:** 10.1186/s12859-014-0366-2

**Published:** 2014-11-19

**Authors:** Chien-Chi Lo, Patrick S G Chain

**Affiliations:** Bioenergy and Biome Sciences Group, Los Alamos National Laboratory, Los Alamos, NM 87545 USA; Genome Science Group, Bioscience Division, Los Alamos National Laboratory, Los Alamos, NM 87545 USA

**Keywords:** Quality control, Trimming, Next generation sequencing analysis, Data preprocessing

## Abstract

**Background:**

Next generation sequencing (NGS) technologies that parallelize the sequencing process and produce thousands to millions, or even hundreds of millions of sequences in a single sequencing run, have revolutionized genomic and genetic research. Because of the vagaries of any platform’s sequencing chemistry, the experimental processing, machine failure, and so on, the quality of sequencing reads is never perfect, and often declines as the read is extended. These errors invariably affect downstream analysis/application and should therefore be identified early on to mitigate any unforeseen effects.

**Results:**

Here we present a novel FastQ Quality Control Software (FaQCs) that can rapidly process large volumes of data, and which improves upon previous solutions to monitor the quality and remove poor quality data from sequencing runs. Both the speed of processing and the memory footprint of storing all required information have been optimized via algorithmic and parallel processing solutions. The trimmed output compared side-by-side with the original data is part of the automated PDF output. We show how this tool can help data analysis by providing a few examples, including an increased percentage of reads recruited to references, improved single nucleotide polymorphism identification as well as *de novo* sequence assembly metrics.

**Conclusion:**

FaQCs combines several features of currently available applications into a single, user-friendly process, and includes additional unique capabilities such as filtering the PhiX control sequences, conversion of FASTQ formats, and multi-threading. The original data and trimmed summaries are reported within a variety of graphics and reports, providing a simple way to do data quality control and assurance.

**Electronic supplementary material:**

The online version of this article (doi:10.1186/s12859-014-0366-2) contains supplementary material, which is available to authorized users.

## Background

With the concurrent increases in total data output and decreases in costs associated with sequencing technology over the last few years, sequencing has now become routine, with many small to large laboratories or institutes generating billions (GB) to trillions (TB) of base pairs of data using one or more of today’s high throughput, small read platforms (Ion, Illumina, SOLiD). However, each technology, specific platform, and even each sequencing run can display a unique error profile, whose provenance is from a combination of incomplete chemical reactions and stochastic errors in the biological processes of both library creation and the specifics of the sequencing reactions, coupled with occasional issues in detecting the signal from polymerase extension [1,2]. Because most sequencing analysis software assumes accurate data as input, these errors can sometimes bias or even grossly mislead biological interpretations. While there is no standard method to deal with low quality data, getting rid of poor quality data can in general, improve the result of downstream analysis [3].

A number of tools have been developed to assess and summarize the quality of a given sequencing run. FastQC (bioinformatics.babraham.ac.uk/projects/fastqc/) for example, provides a rapid quality check by subsampling from the total sequence dataset. While this type of initial assessment may be useful for determining issues within the library creation and sequencing pipelines, for downstream analysis it is preferable to attempt to preprocess all of the data to remove likely or obvious errors. A few applications already exist that provide both quality checks and preprocessing capabilities for today’s NGS reads, however, they all differ, both in convenience and in capability. For example, the SolexaQA package [3] comes in three command-line components that need to be run successively to perform both quality control (QC) and trimming, while PRINSEQ [4] is available as either a command-line program or via a web-interface. The FASTX-Toolkit (http://hannonlab.cshl.edu/fastx_toolkit/) in contrast, is a collection of command-line tools that are also available via the Galaxy platform [5,6], but they must be developed into custom pipelines for data QC and trimming.

In addition to differences in user interfaces, all tools are currently missing some features that limit their utility. For example, PRINSEQ was initially designed for preprocessing 454 Pyrosequencing data, and while it has been extended to function on single Illumina datasets, it is not efficient on large datasets in terms of runtime and memory usage for a complete statistical report. The trimming component of SolexaQA does not have the ability to remove (filter) unwanted reads, such as those with low complexity, and requires an additional step to pair the trimmed reads for downstream applications. In addition, while it can auto-detect the FASTQ format, the quality check component is restrictively strict on the sequence ID format that creates problems for applying it to data from other sequencing platforms. Lastly, FASTX-Toolkit can only process one FASTQ variant (the ASCII offset 64 format) and requires further processing to link paired reads (a feature not provided in FASTX). In addition to these tool-specific issues, none of the aforementioned programs take advantage of multiprocessors, which are common even on everyday laptops, therefore, these tools can become severely time-consuming and possibly limiting, given the very large datasets being generated today. The constant increase in sequencing throughput, and the democratization of sequencing to laboratories less experienced in sequencing, demands more efficient and adaptable software solutions.

In order to address some of the limitations listed above, we developed FaQCs (FastQ Quality Control Software), an efficient, parallelized, all-in-one program that provides a simple yet tunable, user-friendly method to perform data quality monitoring of sequencing runs, coupled with data quality trimming. Both textual and graphical reports for both the input next generation sequencing data and the processed results are generated by default, with a few optional functionalities and outputs. Table 1 compares the full list of features of this new program with existing quality control and preprocessing tools. The program is publicly available at https://github.com/LANL-Bioinformatics/FaQCs, as both a standalone version and as a galaxy module that can be installed on any local Galaxy instance [6].

**Table 1:** **Features comparison for various QC tools**

**Features**\**Tools**	**FaQCs**	**FastQC v0.10.0**	**FASTX**-**Toolkits v0.0.13**	**PRINSEQ lite v0.20.4**	**SolexaQA v2.2**
**3’ end quality trimming**	**Yes**	No	**Yes**	**Yes**	**Yes**
**5’ end quality trimming**	**Yes**	No	No	**Yes**	**Yes**
**Cross quality spike trimming** ^**%**^	**Yes**	No	No	No	No
**Adapter/Primer trimming**	**Yes**	No	**Yes**	No	No
**PhiX filtering**	**Yes**	No	No	No	No
**Low complexity filtering**	**Yes**	No	**Yes**	**Yes**	No
**“N” base filtering**	**Yes**	No	**Yes**	**Yes**	No
**Length filtering**	**Yes**	No	**Yes**	**Yes**	Yes*
**Sequence duplication filtering**	No	No	**Yes**	**Yes**	No
**Kmer content/rarefaction**	**Yes**	**Yes**	No	No	No
**Graphic quality report output**	**Yes**	**Yes**	Yes*	Yes*	**Yes**
**GC (nucleotide) content distribution**	**Yes**	**Yes**	**Yes**	**Yes**	No
**Fastq format conversion**	**Yes**	No	No	No	No
**Multiple fastq inputs**	**Yes**	**Yes**	No	No	**Yes**
**Process paired-end data**	**Yes**	No	No	**Yes**	Yes*
**Accept fastq variants format**	**Yes**	**Yes**	No	**Yes**	**Yes**
**Support compressed gzip input data**	**Yes**	**Yes**	No	**Yes**	No
**Multi-threaded**	**Yes**	**Yes**	No	No	No
**Stand-alone tool/Command line**	**Yes**	**Yes**	**Yes**	**Yes**	**Yes**
**Web-interface**	**Yes** ^**#**^	**Yes**	**Yes** ^**#**^	**Yes** ^**$**^	No

^%^FaQCs records a minimum of five bases of quality scores from both ends.

*Uses a separate program/script to generate the result.

^#^Module for Galaxy platform.

^$^A separate web version is required.

## Implementation

**Figure 1:**
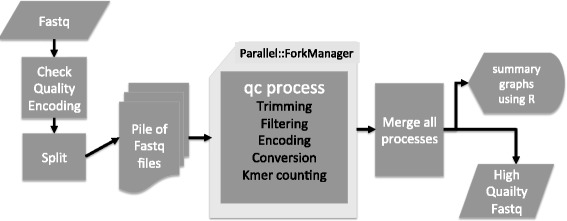
**FaQCs Flowchart.** FASTQ files input are first checked for the format of quality encoding, then split into a set (pile) of files which are subsets of the original input. Each file is processed independently and managed using the Parallel::ForkManager Perl module. A global data structure is used to store results returned from each parallel process. All reports are merged and a processed FASTQ file along with a series of detailed graphics are output in PDF format.

The FaQCs program is written primarily in Perl, takes as input sequence read files in any FASTQ format, and outputs the trimmed and filtered reads, along with summaries of the data that include textual and graphical outputs, which are also combined into a single user-friendly PDF file. FaQCs requires R [7] to be installed in order to generate the graphical outputs and summary PDF file, and also requires the Perl Parallel::ForkManager and String::Approx Modules from CPAN (the Comprehensive Perl Archive Network) to allow parallel processing and adapter trimming. An optional k-mer counting application requires the Jellyfish program [8], but this is the sole other dependency. The data flow and processing steps are shown in Figure 1.

FaQCs can process one or more Illumina-style FASTQ files as input, and these can be unpaired sequence reads, paired sequence reads in two separate files, or mixed paired and unpaired reads. FASTQ files contain information about each base call, as well as its associated quality score, encoded in ASCII characters. As the technologies progressed over the years, and users became more adept at handling these new large data files, a number of FASTQ variants have emerged that utilize different encoding ASCII ranges. This has had a severe impact on data interpretation, has confused many end-users of the data, and continues to create problems when using specific downstream analysis software [9]. We have therefore incorporated as a built-in default feature the ability to detect the quality encoding format and have the data converted, if needed, to Sanger-style quality format, which is now accepted by the majority of downstream data analysis software. FaQCs also supports the GZIP compression algorithm, allowing direct processing of compressed data.

Because today’s NGS platforms can generate a tremendous amount of data, we included parallel processing as part of FaQCs functionality. To process the input data in parallel, FaQCs uses the Parallel::ForkManager Perl module to control the sub-processes. The input dataset is initially split by default into multiple files of 1 million reads each, but users can tune this parameter via a command line flag. Each split file is independently run through the QC process, controlled by ForkManager in parallel. If the number of files is bigger than the specified processor number, it sequentially fills the next available processor that has presumably finished performing QC on a previous file. FaQCs reads through each subset of data in each sub-process and keeps all statistics in memory. Once a sub-process finishes, a global data structure retains and combines the statistical outputs of each sub-process and the memory from that sub-process is made available for other tasks. When all sub-processes are complete, data matrices are written to text files, which are then used to generate graphical reports.

For convenience, FaQCs provides a processing mode to perform only quality monitoring on a subset of the input dataset without trimming and filtering, similar to the behavior of FastQC, however this is not the default setting. FaQCs can utilize either a trimming algorithm similar to BWA [10] (default), or optionally, a simple hard cutoff on quality score. One novel feature is that FaQCs implements trimming not only from the 3’ end of the reads, but will also trim from the 5’ end. In brief, the BWA-style trimming converts the ASCII characters to decimal numbers and finds the position in the read where trimming will end (argmax) based on the following equation:

1 $$ \underset{x\in \left\{1,2,\dots, l\right\}}{ \arg \max }{\displaystyle {\sum}_{i=x}^l\left({Q}_u-{Q}_i\right)}\;\mathrm{if}\;{Q}_u>{Q}_u $$

where l is the read length and Q_u_ is the user-defined quality threshold, and trimming ends after the summation of Q_u_ – Q_i_ becomes negative. The default value for Q_u_ is Q =5, since most bases with a quality score of 4 or lower have been shown to be erroneous [2]. To deal with cases where one or two bases near the ends display a spike in Q score but then return to a poor Q value (Q <5), we implemented a novel function which forces the algorithm to record at lease five bases of quality scores from both the 5’ and 3’ ends, in addition to 2 further positions to account for these possible sudden spikes in quality.

In addition to identifying regions of low quality for trimming, we have added a variety of parameters to further trim and filter (i.e. remove) reads. The sequences can be adapter- and primer-trimmed given user input on those specific sequences. After trimming, by default, reads with two continuous ambiguous (N) bases are removed, together with those under 50 bases or with >85% low complexity (mono-/di-nucleotide repeat sequence). The following parameters allow users to tailor their trimming and filtering criteria to their needs: minimum length cutoff, minimum average quality score, maximum number of continuous ambiguous (N) bases, and maximum percentage of low complexity bases, matches to phiX.

**Figure 2:**
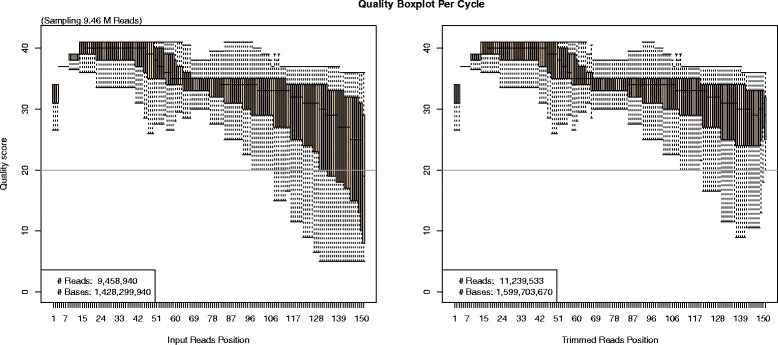
**Boxplot graph for the quality scores.** Rectangular boxes show the Inter-quartile Range (IQR). The end of the whiskers shows outliers at max 1.5*IQR. Horizontal lines in the box are median values at each bp position. There is a horizontal line at quality 20 indicating the predicted per base error rate of 1/100. For easy comparison, FaQCs generates two boxplots side by side where the left panel is the boxplot of the raw reads and the right represents the processed reads. This is but one set of figures generated in the final PDF report (see Additional file 1: Figure S1).

After running one or more FASTQ files, the output consists of: 1) the trimmed and filtered sequences in paired and/or single end read FASTQ files (depending on input); 2) a summary PDF report and text file on the raw and trimmed data. The PDF report, using default parameters, includes eight sections: 1) summary statistics of the raw and trimmed data, 2) read length histograms of raw and trimmed data, 3) nucleotide content histograms, 4) nucleotide content per base graphs, 5) average quality per read histogram, 6) quality boxplots per base, 7) quality 3D plot of base position vs. quality score vs. frequency, and 8) quality score histogram (Additional file 1: Figure S1). Each graph provides users with a unique perspective on the data for thorough quality assessment. For example, Figure 2 shows the quality boxplot output per base position of a MiSeq dataset both before and after the QC process.

**Figure 3:**
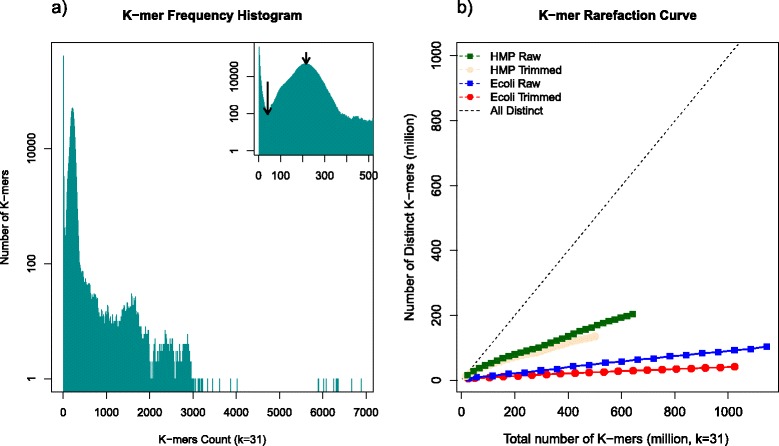
**Plots from of k-mer profiling. a)** K-mer frequency histogram of *E.coli* MiSeq dataset shows an obvious peak k-mer coverage near 216X (small arrow, inset figure) and a minimum inflection point at ~41X (long arrow, inset figure). The k-mers below than the minimum inflection point are due to sequencing artifacts and errors. The other small peaks typically indicate repeats in the genome. **b)** K-mer rarefaction curve shows a reduction of k-mers when trimming. The blue and red soild lines are the k-mer rarefaction curves of raw and trimmed *E.coli* MiSeq data, respectively. The green and beige solid lines are k-mer rarefaction curves of raw and trimmed data of the HMP Mock data, respectively. The dashed line represents the baseline where all observed k-mers are distinct.

FaQCs provides additional optional functionalities found in few, or none of the other existing sequence data trimming utilities. For example, FaQCs can implement Jellyfish, a fast k-mer counting tool, to allow visual interpretation of k-mers and their abundance (Figure 3), which can help predict the completeness of coverage of the sequencing target. By default, FaQCs performs k-mer counting on a subset (ten) of the split files, and merges the ten k-mer frequency profiles. The output of k-mer counting includes two additional graphs that are added to the final PDF. One of the graphs is a k-mer frequency histogram (Figure 3a), which displays the distribution of k-mer abundances; i.e. how many k-mers (Y axis) are represented at what frequency (X axis). *De novo* assembly of single genomes can use the k-mer abundance peaks to estimate the fold coverage of a target genome; and users can use this information to better estimate the target genome size given the number of different k-mers surrounding the peak abundance. The other output is a k-mer rarefaction curve that users may use to judge whether the sequencing effort is sufficient if sequencing a single organism (Figure 3b). While useful, k-mer counting is provided only as an optional parameter, primarily because this step consumes non-trivial additional CPU memory and time, and may not always be a necessary calculation (outside of the use cases described above).

For further user-friendliness, we have implemented FaQCs as a Galaxy module with detailed information about its operation. The Galaxy interface allows easy manipulation of sequencing data as part of larger workflows and/or via point and click, if this is preferred to running the program via command line or incorporating this tool into a larger program.

## Results and discussion

### Performance

To illustrate the program’s capabilities, we consider different types of datasets, summarized in Table 2. To allow a fair comparison between the single-step FaQCs and the other available, multi-step read-trimming quality control tools (PRINSEQ, SolexaQA, FASTX), we complied the serial steps of each tool and used the same quality thresholds for the read preprocessing of a few different samples. Because FastQC does not have trimming and filtering features, it was not included in this comparison. All runs were submitted to a cluster node equipped with Intel(R) Xeon(R) CPU X5675 @ 3.07GHz, 24 processors and 64G memory.

**Table 2:** **Data analyzed in this study**

**Dataset**	**Source**	**Platform**	**Pre-QC**				**After-QC**	
			**# of Reads (bases)**	**Average length (bp)**	**Average quality**	**# of Reads (bases)**	**Average length (bp)**	**Average quality**
***Escherichia coli*** **MG1655**	Illumina*	Illumina MiSeq	11458940 (1730299940)	151.00	32.32	11239533 (1599703670)	142.33	33.70
***Providencia stuartii*** **33672**	NCBI SRA SRX687104	Illumina MiSeq	26654456 (2692100056)	101.00	29.63	20586466 (2006232265)	97.45	33.08
***Escherichia coli*** **Outbreak strain**	NCBI SRA SRX067313	Ion Torrent PGM	783097 (79769722)	101.86	18.32	435634 (25445098)	58.41	26.03
**HMP Mock**	NCBI SRA SRX055380 SRX055381	Illumina GAII	14494884 (1087116300)	75.00	25.13	13502777 (922535214)	68.32	28.40

*http://www.illumina.com/systems/miseq/scientific_data.ilmn.

Table 3 shows the computational performance of all tools on an isolate genome dataset and a metagenome dataset. While taking only one CPU process, PRINSEQ took over 20x more memory and 1.5x Wallclock time than FaQCs. This is primarily due to PRINSEQ’s effort to identify duplicate reads within the entire dataset. SolexaQA consumed even less memory since it only processes a subset of the data for the quality report. FASTX however, outperformed all others, due in part due to its implementation in C++ and resulting fine control on memory usage and overhead on CPU processors. However, FASTX also does not track paired end reads, and requires multiple modules to be run on both the raw and trimmed reads to obtain pre- and post-trimming statistics.

**Table 3:** **The comparison of the computational performance using** ***E.coli*** **MG1655 MiSeq dataset and HMP Mock GAII dataset**

***E.coli*** **MG1655 MiSeq**	**FaQCs**				**FASTX**	**PRINSEQ**	**SolexaQA**
**Parallel Process**	1	4	8	12	1	1	1
**Memory**	192.60 M	354.97 M	568.36 M	739.09 M	74.70 M	4.05G	85.87 M
**Wallclock Time**	1:59:28	0:36:34	0:26:16	0:14:16	0:31:15	3:03:29	4:20:55
**CPU**	1:58:22	1:59:59	2:02:43	2:03:10	0:30:27	2:59:57	4:13:07
**HMP Mock**	**FaQCs**				**FASTX**	**PRINSEQ**	**SolexaQA**
**Parallel Process**	1	4	8	12	1	1	1
**Memory**	189.99 M	341.71 M	573.69 M	738.57 M	39.08 M	16.46G	38.48 M
**Wallclock Time**	1:16:35	0:25:12	0:17:09	0:12:54	0:17:04	2:47:51	1:29:03
**CPU**	1:14:53	1:14:49	1:21:49	1:24:22	0:16:21	2:43:26	1:28:23

To take advantage of multiprocessors that can be found in most computers, we tested the multi-threading capability of FaQCs using 4, 8 and 12 parallel processers. FaQCs performs up to 6 ~ 8x faster. As expected, the increase in number of processors is proportional to the increase in memory consumed, since additional memory is required to store the read information with each additional split file. Despite this however, for the dataset tested, FaQCs can complete in less than 15 minutes with 12 parallel processors, and given the memory consumption, can be readily run on a many of today’s laptop computers.

### Expected effect on downstream data analyses

Because it has already been shown that trimming and filtering sequencing reads benefit downstream analysis [3], here we only briefly provide examples of *de novo* assembly and read mapping effects of FaQCs data processing (Additional file 2: Tables S1 and S2), using raw data and the matching reference genome(s). After preprocessing four example datasets for three isolate genomes and a metagenome, FaQCs retained a range from 77.23% to 98.09% of the original input reads and 74.52% to 92.45% of the original total bases. The trimmed and raw reads were normalized by the number of reads, and were submitted to Velvet (version 1.2.07) [11], Newbler (version 2.6) [12] or IDBA_UD (version 1.1.1) [13] for *de novo* assembly of Illumina and Ion Torrent data. Differences between the assembly and the reference were obtained using NUCmer (version 3.07) [14] to map the contigs to the appropriate reference genome. The reads were also mapped to the reference genome using BWA (version 0.6.2) [10] and SNPs were then called by SAMtools (version 0.1.18) [15], followed by filtering of those that were located in repeatitive regions.

Because *de novo* assembly results can vary depending on the parameters used, we explored a Velvet k-mer spectrum from 63 to 119 for the isolate genome, but with all other default parameters set to default. We summarize the results in Additional file 3: Figure S2, comparing the relative assembly size compared with the reference genome and the number of single nucleotide polymorphisms. The Newbler assembly of Ion Torrent data from isolates, and IDBA_UD assembly of metagenome data are summarized in Additional file 2: Table S1. Almost invariably, the trimmed reads produced better results (assembly most consistent with the reference, and fewer SNPs) when compared with untrimmed data, consistent with the expectation that trimming poor quality improves assembly results [3]. Furthermore, Additional file 2: Table S2 indicates that trimming also improves read-mapping based analyses, the trimmed reads have a greater proportion of reads mapped to the reference sequence and fewer SNPs would be reported (i.e. fewer false positive SNPs). These improvements are seen even with this high quality dataset, when only 1.91% of the reads were discarded (7.55% nucleotide bases); the benefit of trimming is expected to be much more drastic with datasets of lower quality.

### An integrated k-mer counting utility

Users can activate an optional k-mer calculation function that uses Jellyfish, for a rarefaction curve and a k-mer histogram plot to be provided in the final report. By default, the function is not on because it significantly prolongs the execution time and increases memory usage. However, the result of k-mer counting can provide users with the ability to estimate genome size manually and evaluate whether the sequencing effort is sufficient or not.

The k-mer histogram displays the number of k-mers (the number of different k-mer words) versus the k-mer count (i.e. the number of times that particular k-mer is observed), and can assist users to interpret their data. An isolate genome ideally will have one major k-mer coverage peak (i.e. most k-mers in the genome will be observed a similar number of times), with possibly smaller peaks of more frequently observed k-mers representing repeat regions within the genome. Using the *E.coli* MiSeq dataset, an obvious k-mer coverage peak at 216 fold coverage can be observed, with a minimum infection point at 41 fold coverage (Figure 3a). K-mers below the minimum inflection point are due to sequencing artifacts and errors.

For an isolate genome, one can use the largest k-mer peak to estimate the average nucleotide fold coverage using the formula:

2 $$ \mathrm{Fold}\;\mathrm{Coverage}=\left(\mathrm{peak}\;\mathrm{k}\hbox{-} \mathrm{m}\mathrm{e}\mathrm{r}\;\mathrm{coverage}\right)\times \mathrm{L}/\left(\mathrm{L}-\mathrm{K}+1\right) $$

where L is the average read length and K is the k-mer size.

For example, given the average trimmed length of 142.33 bp and k-mer size of 31 for the *E. coli* dataset, the nucleotide fold coverage is estimated as 274X. This differs from actual mapping fold coverage (337X in Additional file 2: Table S2), likely due to the exactness of the k-mer counting function and the allowance for mismatches in the read-mapping procedure. The genome size can also be approximated by using the total number of non-erroneous k-mers (k-mers larger than the minimum left infection point), which in this example is 4,553,316 bp compared with the actual reference size of 4,639,675 bp.

A k-mer rarefaction curve is also provided. In Figure 3b, the rarefaction curve of the isolate dataset is improved after trimming, showing a flatter curve, consisting of the finite k-mers within an isolate genome. Metagenomes have a much larger k-mer composition profile, and the rarefaction curve will continue to climb until the metagenome is sufficiently sampled. The decrease in number of total k-mers observed in both the isolate and metagenome data is an indication of the removal of errors within the data during the QC process. Generally this type of graph is utilized to estimate whether additional sequencing is required or if the sequencing has proceeded as expected.

Considering that the *E.coli* reference genome size is 4,639,675 bp and assuming that all k-mers (k =31) are unique for both strands, the upper bound of unique k-mers should be ~9.2 M. In Figure 3b, the raw untrimmed data shows approximately 100 M distinct k-mers (blue line) while the trimmed data (red line) reduces this substantially to approximately 40 M distinct k-mers. While this is a large improvement, this figure is still four fold higher than expected for the *E. coli* genome, indicating that some sequencing errors still remain within the dataset. A single nucleotide error in the middle of a read can introduce up to 31 unique and distinct k-mers, when K =31. This in turn corresponds to a minimum of 1,036,117 errors remaining in this dataset, or a post-trimming error rate of 0.065%. Therefore, the rarefaction curve even for an isolate may not completely become a plateau, despite trimming, and will depend on the number of reads and the error rate post-trimming. We therefore suggest using both the k-mer histogram together with the rarefaction curve as well as the other quality statistics to interpret one’s data.

## Conclusions

We present FaQCs, a program that provides a rapid and parallelizable means to remove low quality data from large NGS data files, and provide users with adequate outputs to better interpret their data. This new quality control and quality assurance tool is highly flexible in terms of input, with built-in format detection, allows a number of read-filtering and trimming features, and provides user-friendly summary statistics and graphical outputs to allow in-depth assessment of the data. This tool is also implementable within the Galaxy environment. The resulting trimmed output can yield improvements in downstream analyses, including SNP calling and *de novo* sequence assembly. An integrated k-mer counting option can also be used to estimate genome size, and can allow the user to evaluate whether any additional sequencing effort required.

## Availability and requirements

**Project name:** FaQCs

**Project home page:**https://github.com/LANL-Bioinformatics/FaQCs

**Operating system(s):** Platform independent with primary UNIX support

**Programming language:** Perl and R

**Other requirements:** Perl Parallel::ForkManager and String::Approx Modules from CPAN http://search.cpan.org, and Optional requirements, Jellyfish http://www.cbcb.umd.edu/software/jellyfish/

**License:** GNU GPL version 3 or later.

## Additional files

Additional file 1: Figure S1. Output file generated by FaQCs. A) Summary of trimming statistics; B) Read length histogram; C) Nucleotide composition histogram for the reads; D) Per cycle nucleotide composition plot; E) k-mer rarefaction curve; F) k-mer frequency histogram; G) Average read quality histogram; H) Per cycle quality box plot; I) Per cycle, per score frequency plot; J) Average read quality histogram. Additional file 2: Table S1 and Table S2. The *de novo* assembly and read mapping effects of FaQCs data processing. Additional file 3: Figure S2. Comparisons of assembly completeness and assembly SNP error before and after FaQCs data processing.

## Abbreviations

NGS: Next generation sequencing
QC: Quality control
SNP: Single nucleotide polymorphism
CPAN: The comprehensive perl archive network
