# Supplementary material for: Rapid evaluation and quality control of next generation sequencing data with FaQCs
Source: BMC Bioinformatics. 2014 Nov 19;15(1):366. doi: 10.1186/s12859-014-0366-2 (PMC4246454; doi:10.1186/s12859-014-0366-2)
Supplement: Additional file 1: Figure S1. — Output file generated by FaQCs. A) Summary of trimming statistics; B) Read length histogram; C) Nucleotide composition histogram for the reads; D) Per cycle nucleotide composition plot; E) k-mer rarefaction curve; F) k-mer frequency histogram; G) Average read quality histogram; H) Per cycle quality box plot; I) Per cycle, per score frequency plot; J) Average read quality histogram. [file 12859_2014_366_MOESM1_ESM.pdf]

**Supplementary Figure 1:** Output file generated by FaQCs. A) Summary of trimming statistics; B) Read length histogram; C) Nucleotide composition histogram for the reads; D) Per cycle nucleotide composition plot; E) k-mer rarefaction curve; F) k-mer frequency histogram; G) Average read quality histogram; H) Per cycle quality box plot; I) Per cycle, per score frequency plot; J) Average read quality histogram.

A)

## QC stats

| Before Trimming | After Trimming |
| --- | --- |
| Reads: 11458940 | Reads: 11239533 (98.09 %) |
| Total bases: 1730299940 | Total bases: 1599703670 (92.45 %) |
| Reads Length: 151.00 | Mean Reads Length: 142.33 |
| | Paired Reads: 11041676 (98.24 %) |
| | Paired total bases: 1573382551 (98.35 %) |
| | Unpaired Reads: 197857 (1.76 %) |
| | Unpaired total bases: 26321119 (1.65 %) |

---

Discarded reads: 219407 (1.91 %)
Trimmed bases: 130596270 (7.55 %)
- Reads Filtered by length cutoff (50 bp): 201882 (1.76 %)
- Bases Filtered by length cutoff: 5907870 (0.34 %)
- Reads Filtered by continuous base "N" (2): 0 (0.00 %)
- Bases Filtered by continuous base "N": 0 (0.00 %)
- Reads Filtered by low complexity ratio (0.8): 1805 (0.02 %)
- Bases Filtered by low complexity ratio: 254307 (0.01 %)
- Reads Filtered by avg quality (20.0): 15720 (0.14 %)
- Bases Filtered by avg quality: 2331521 (0.13 %)
- Reads Trimmed by quality (20.0): 6522200 (56.92 %)
- Bases Trimmed by quality: 122102572 (7.06 %)

B)

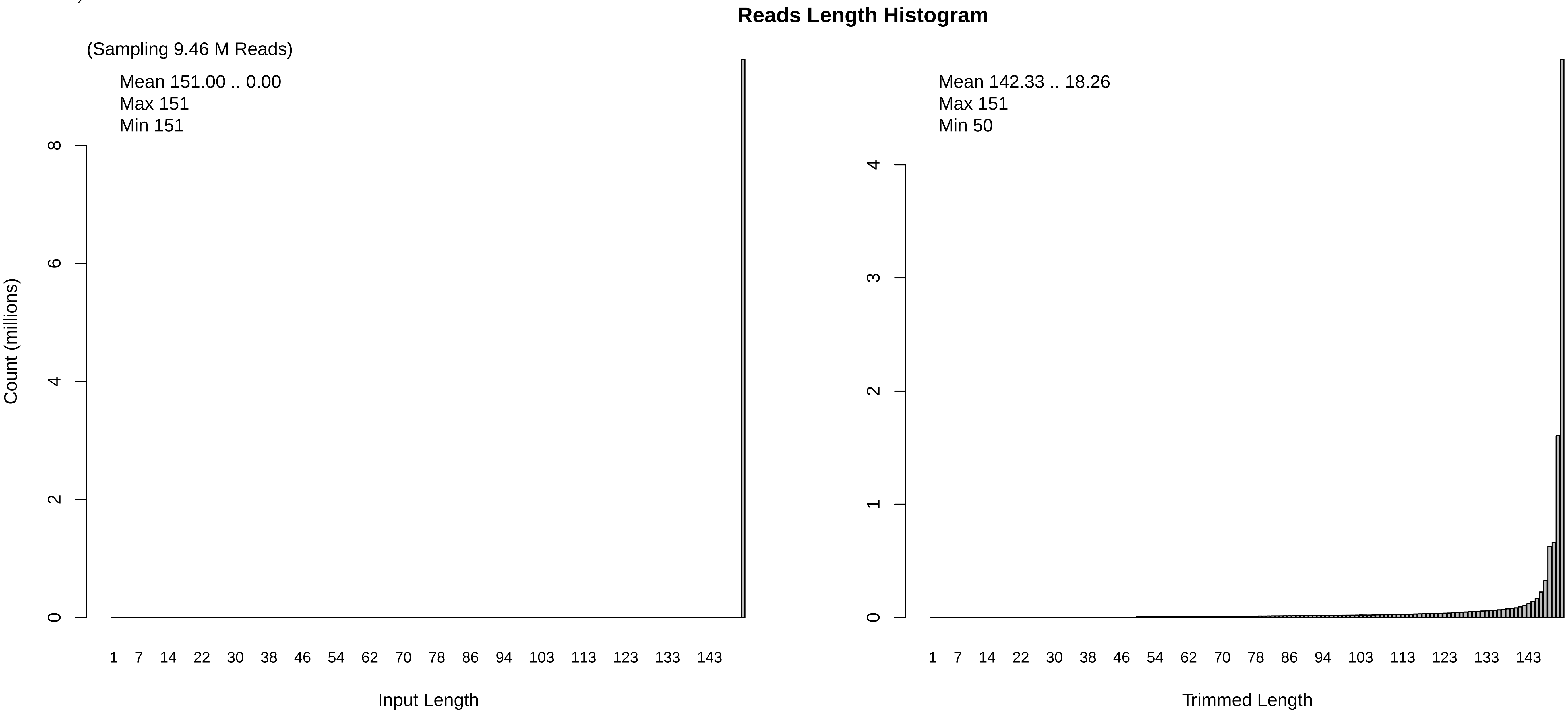
Reads Length Histogram
(Sampling 9.46 M Reads)
Mean 151.00 .. 0.00
Max 151
Min 151
Count (millions)
0
2
4
6
8
1 7 14 22 30 38 46 54 62 70 78 86 94 103 113 123 133 143
Input Length
Mean 142.33 .. 18.26
Max 151
Min 50
0
1
2
3
4
1 7 14 22 30 38 46 54 62 70 78 86 94 103 113 123 133 143
Trimmed Length

C)

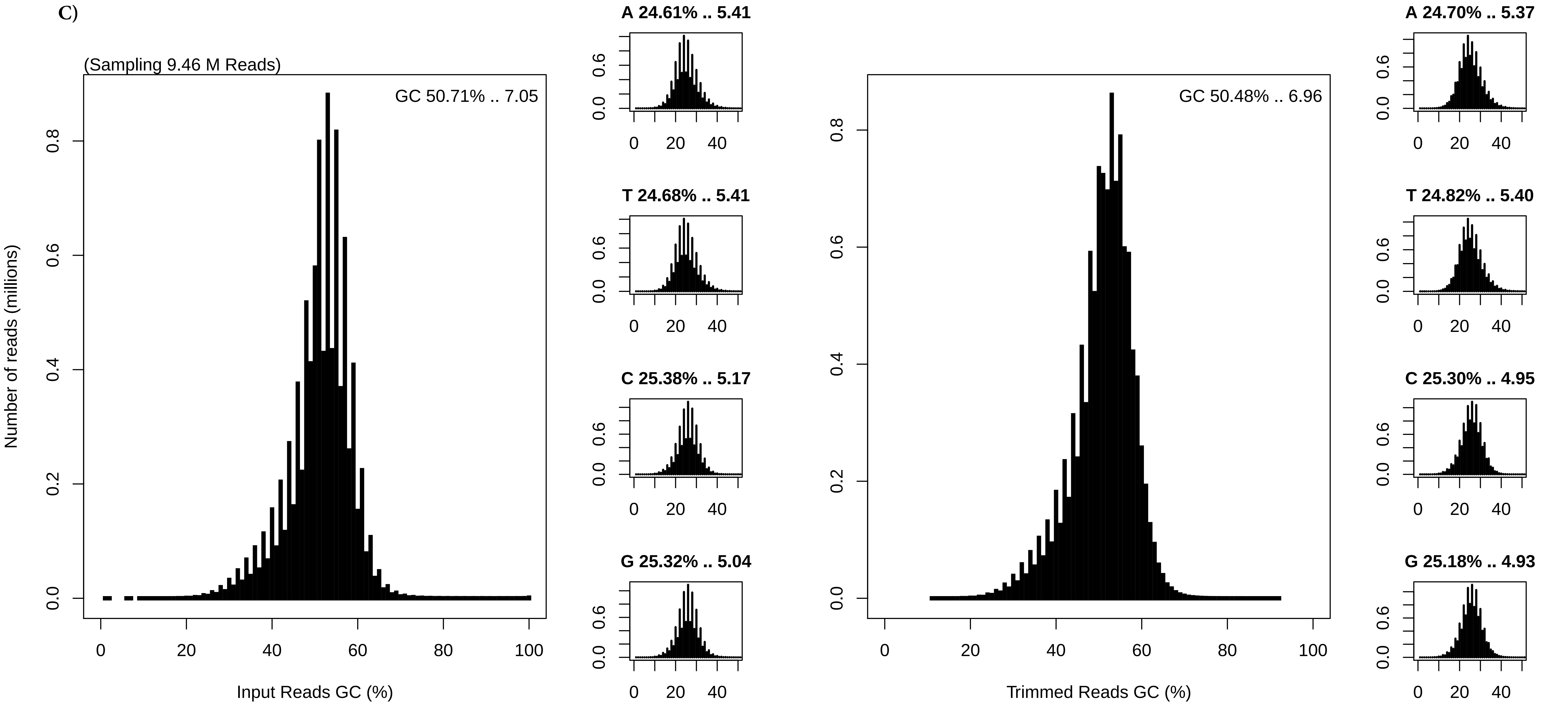
(Sampling 9.46 M Reads)
GC 50.71% .. 7.05
Number of reads (millions)
Input Reads GC (%)
A 24.61% .. 5.41
T 24.68% .. 5.41
C 25.38% .. 5.17
G 25.32% .. 5.04
GC 50.48% .. 6.96
Trimmed Reads GC (%)
A 24.70% .. 5.37
T 24.82% .. 5.40
C 25.30% .. 4.95
G 25.18% .. 4.93

D)

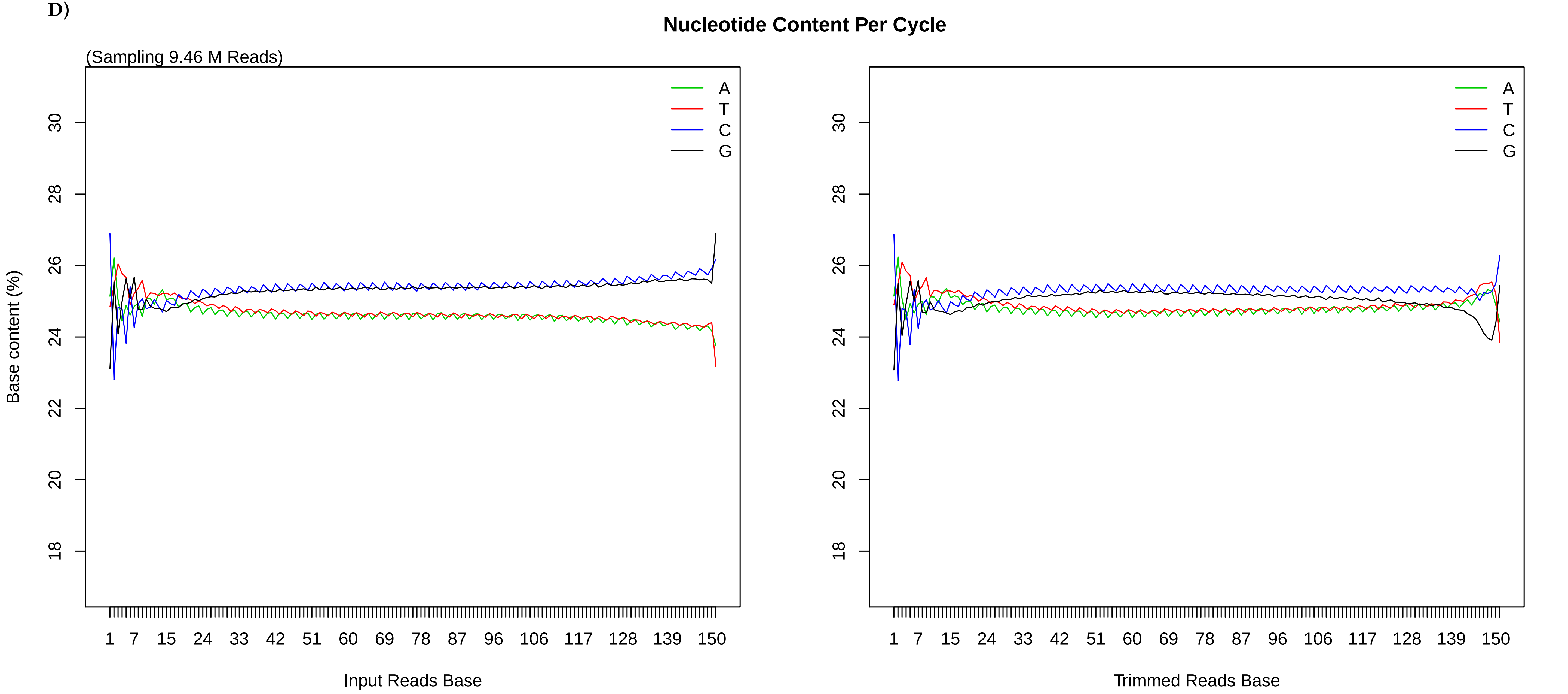
Nucleotide Content Per Cycle
(Sampling 9.46 M Reads)
Base content (%)
A
T
C
G
18
20
22
24
26
28
30
1
7
15
24
33
42
51
60
69
78
87
96
106
117
128
139
150
Input Reads Base
Trimmed Reads Base

E)

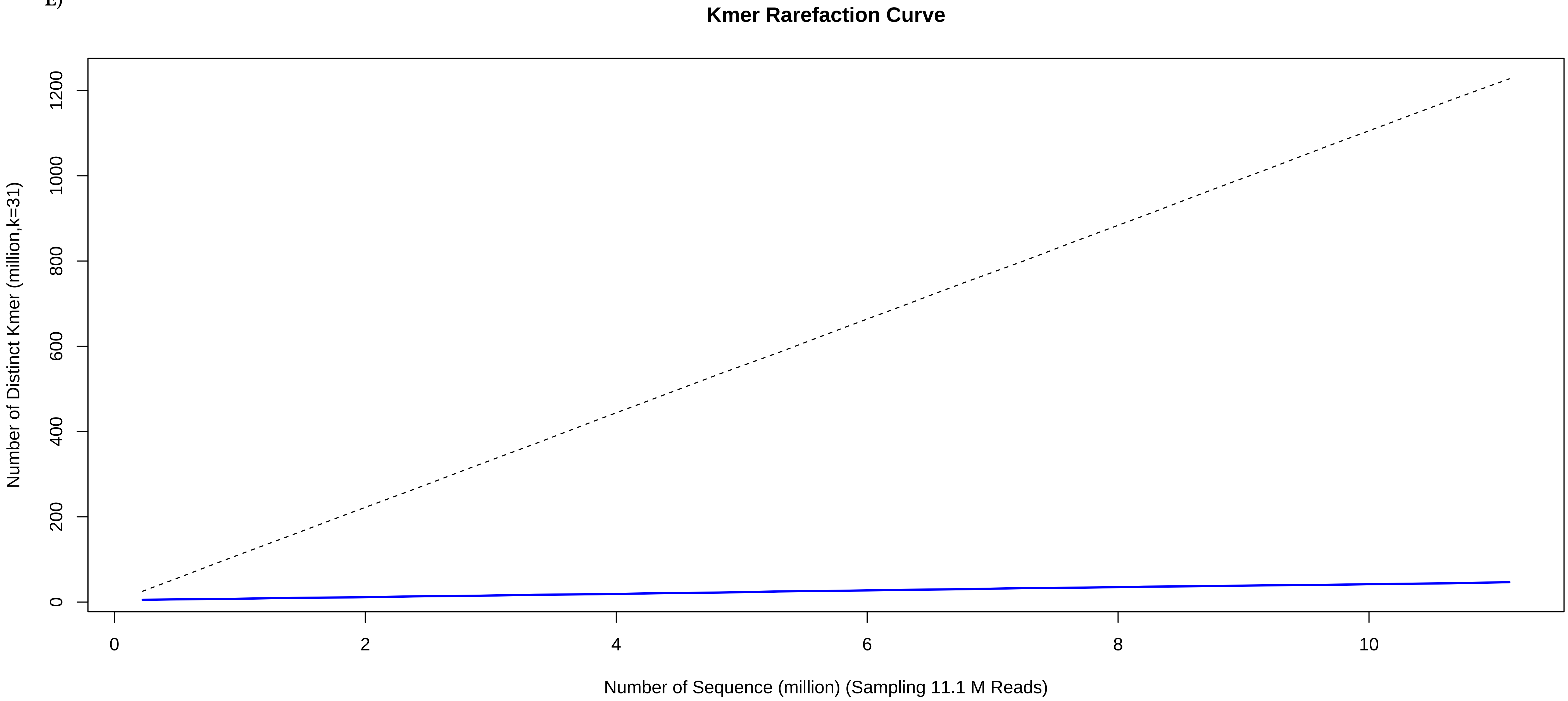

Kmer Rarefaction Curve
Number of Distinct Kmer (million,k=31)
0
200
400
600
800
1000
1200
0
2
4
6
8
10
Number of Sequence (million) (Sampling 11.1 M Reads)

F)

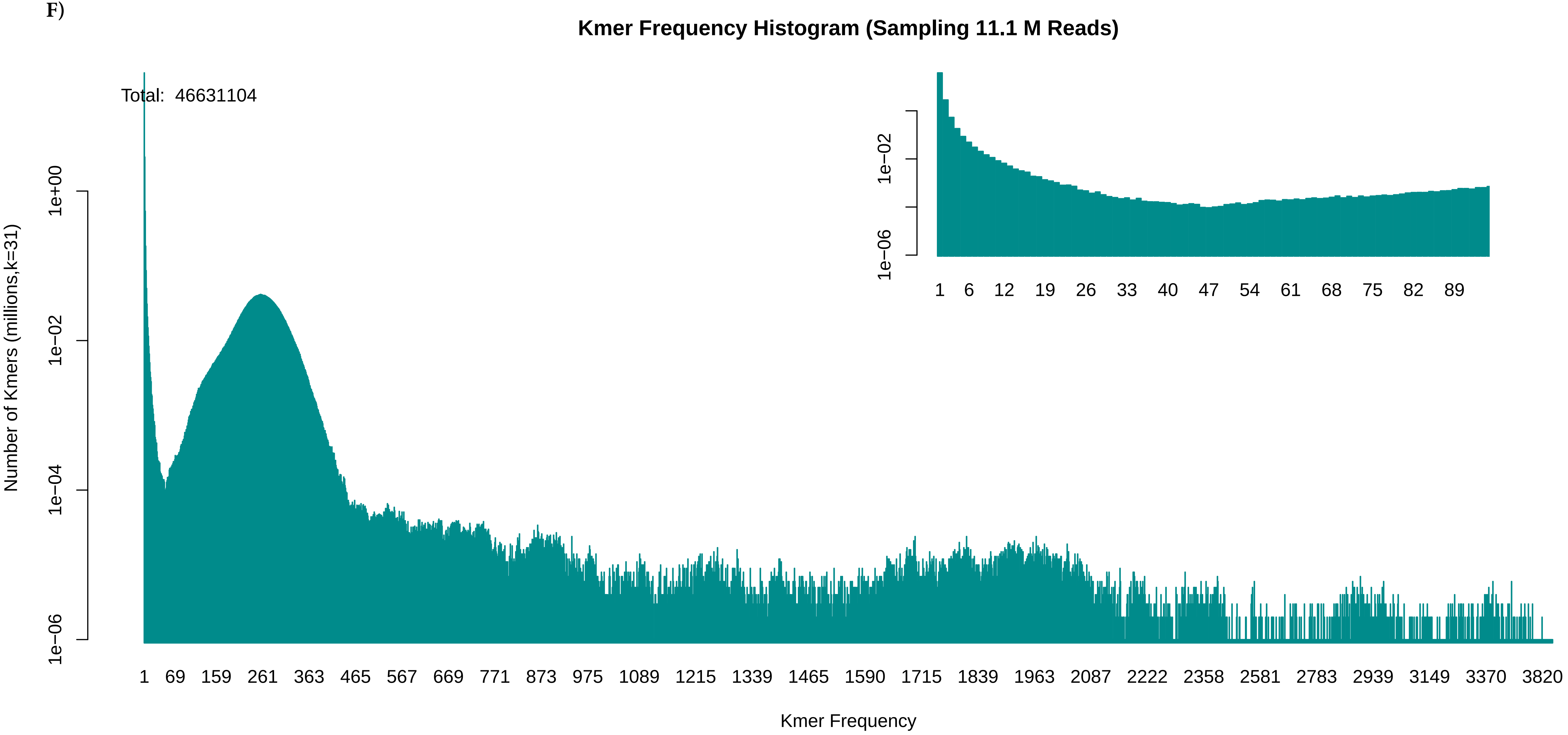
Kmer Frequency Histogram (Sampling 11.1 M Reads)
Total: 46631104
Number of Kmers (millions,k=31)
1e+00
1e−02
1e−04
1e−06
1 69 159 261 363 465 567 669 771 873 975 1089 1215 1339 1465 1590 1715 1839 1963 2087 2222 2358 2581 2783 2939 3149 3370 3820
Kmer Frequency
1e−02
1e−06
1 6 12 19 26 33 40 47 54 61 68 75 82 89

G)

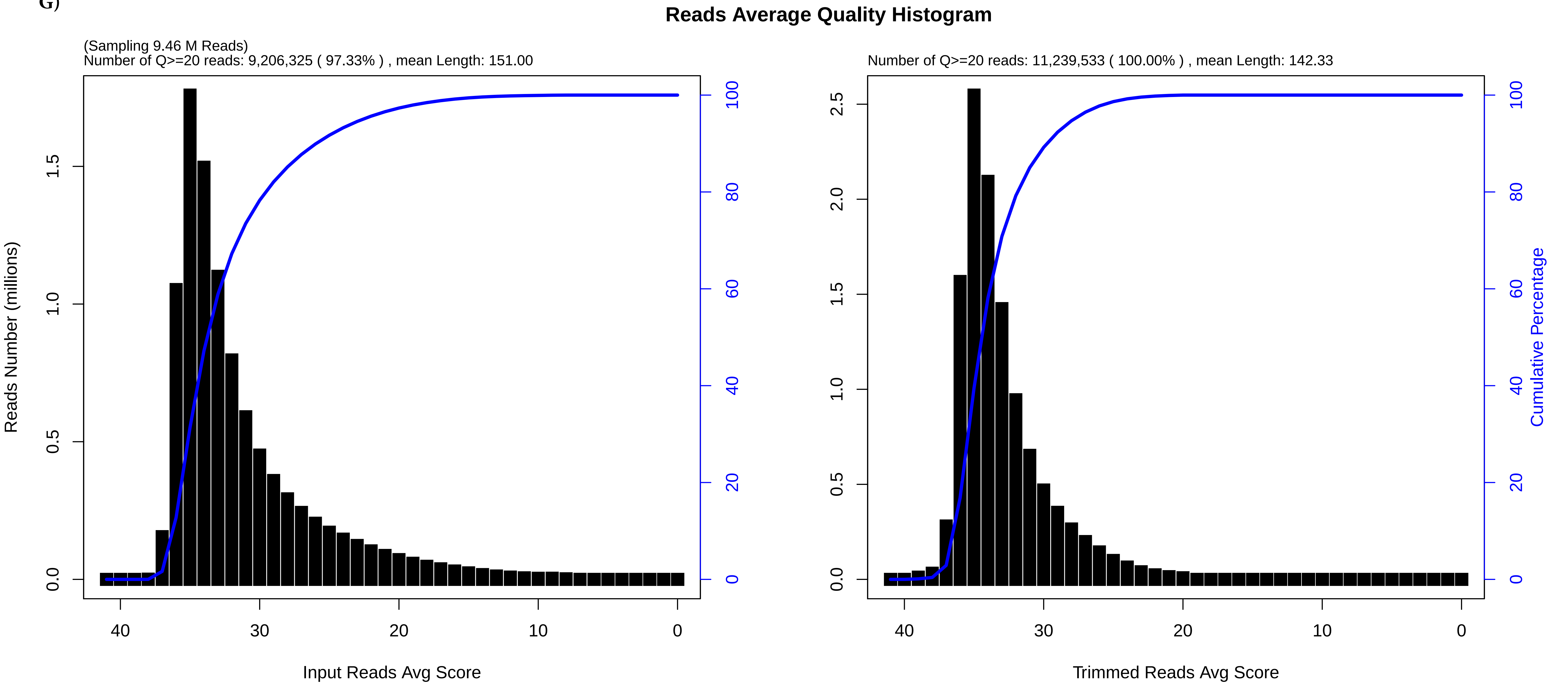
Reads Average Quality Histogram
(Sampling 9.46 M Reads)
Number of Q>=20 reads: 9,206,325 ( 97.33% ) , mean Length: 151.00
Reads Number (millions)
Input Reads Avg Score
Number of Q>=20 reads: 11,239,533 ( 100.00% ) , mean Length: 142.33
Trimmed Reads Avg Score
Cumulative Percentage
0.0
0.5
1.0
1.5
2.0
2.5
40
30
20
10
0
0
20
40
60
80
100

H)

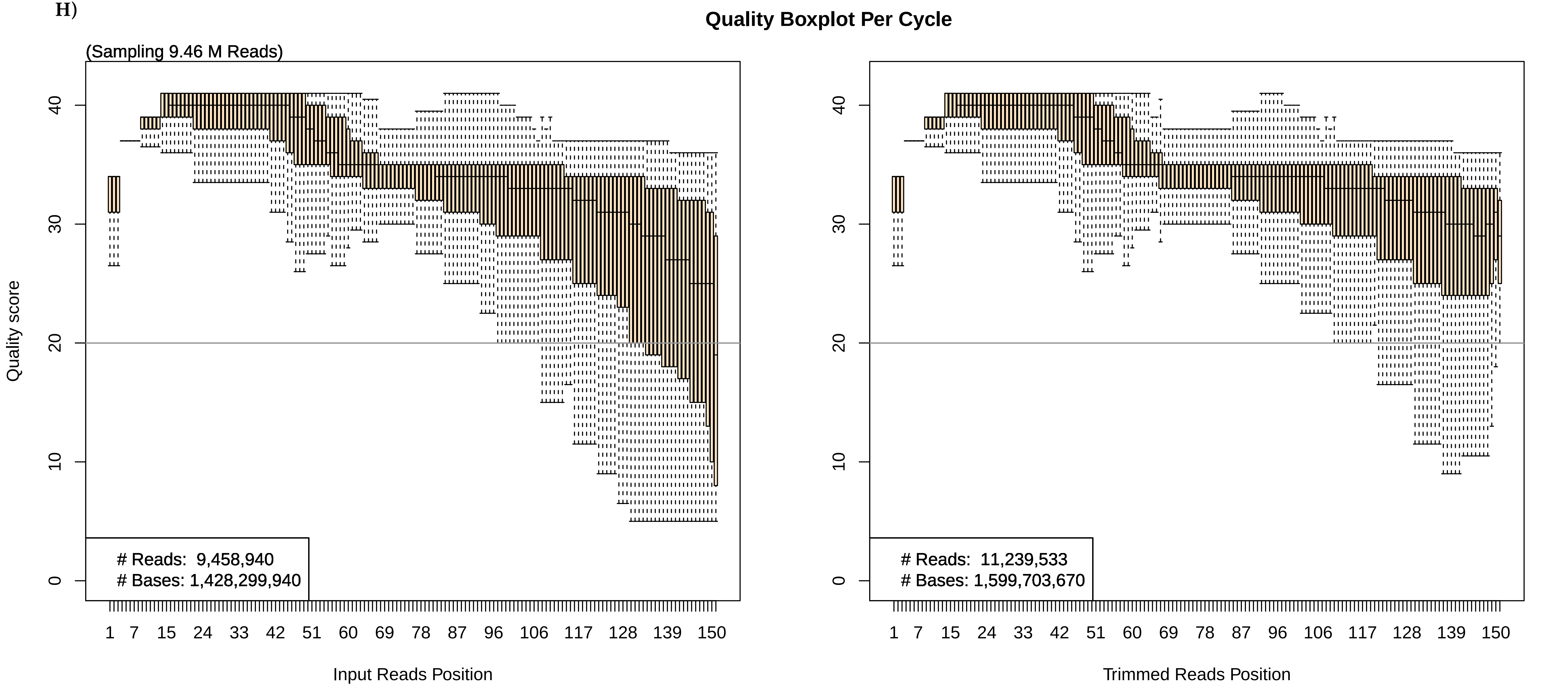

Quality Boxplot Per Cycle
(Sampling 9.46 M Reads)
Quality score
# Reads: 9,458,940
# Bases: 1,428,299,940
Input Reads Position
# Reads: 11,239,533
# Bases: 1,599,703,670
Trimmed Reads Position

I)

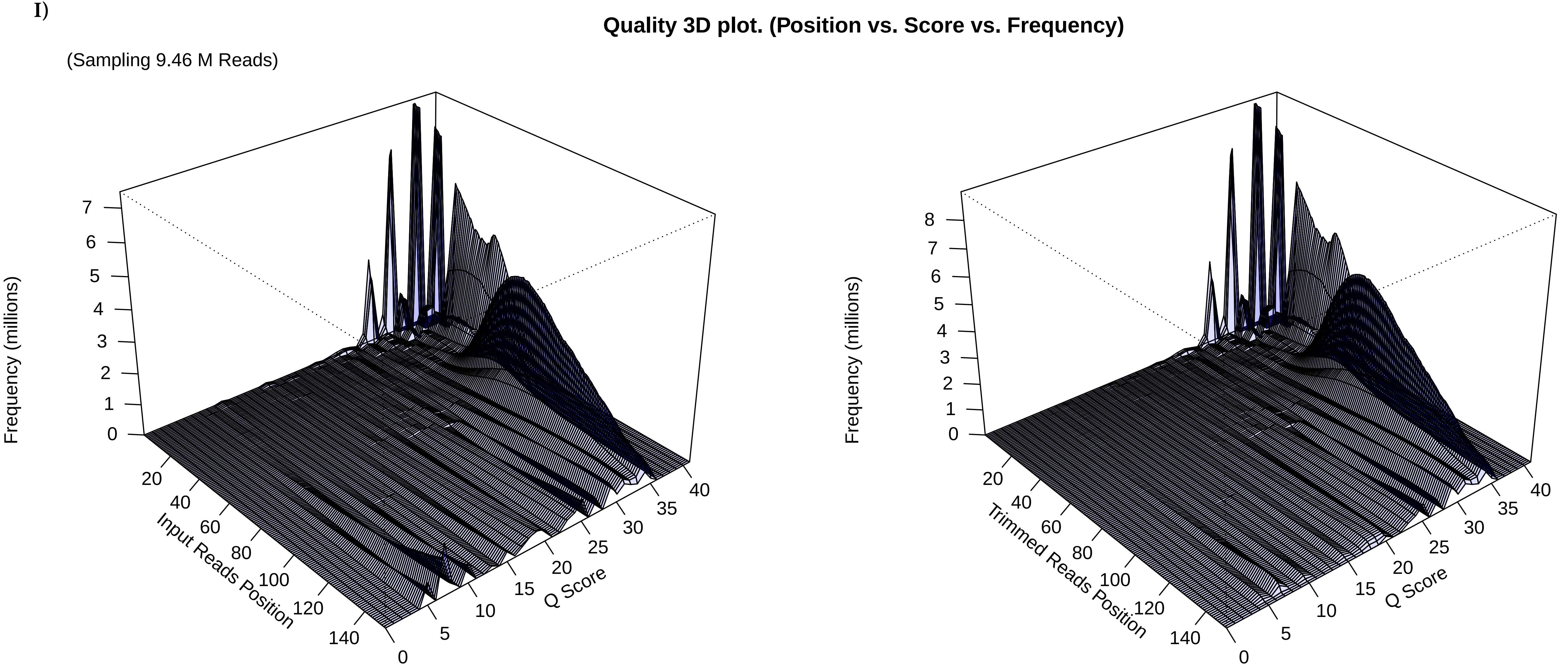
Quality 3D plot. (Position vs. Score vs. Frequency)
(Sampling 9.46 M Reads)
Frequency (millions)
Input Reads Position
Q Score
Frequency (millions)
Trimmed Reads Position
Q Score

J)

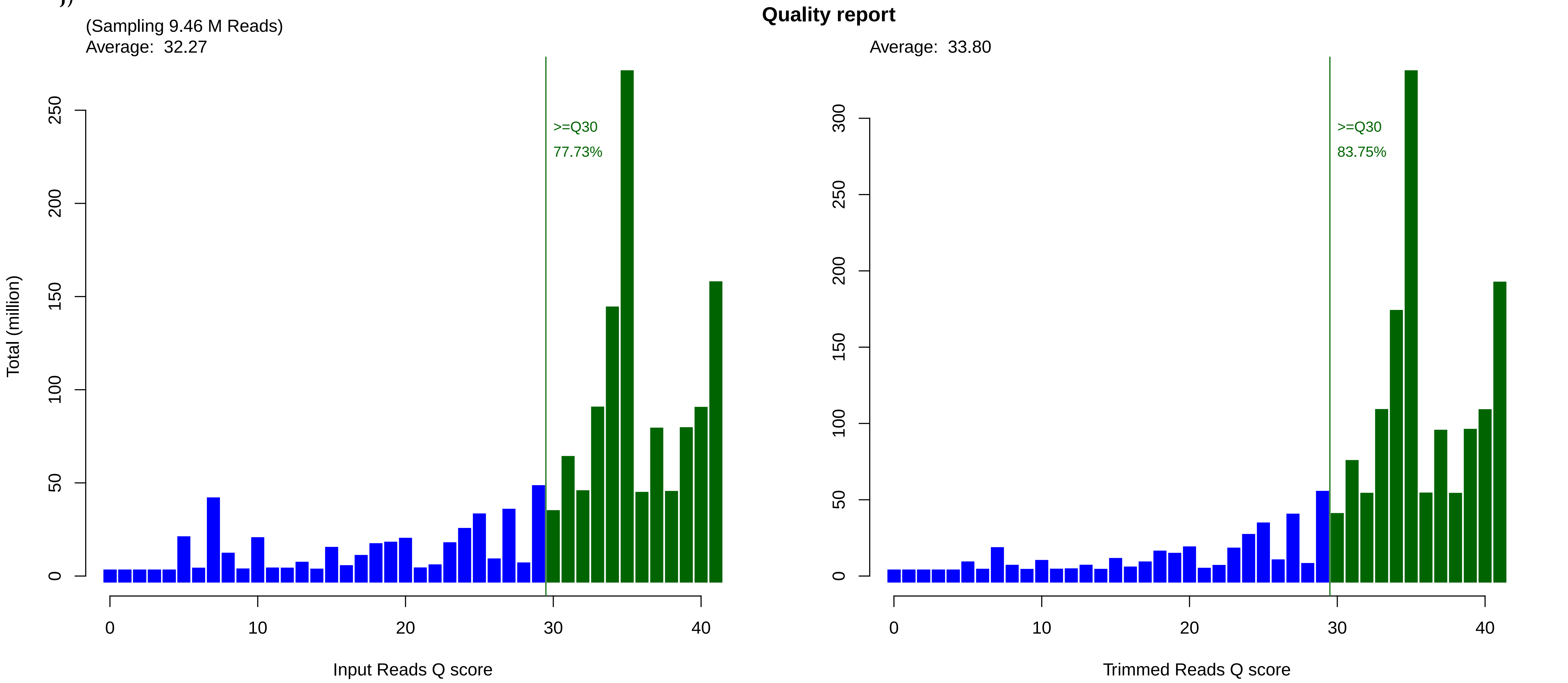
Quality report
(Sampling 9.46 M Reads)
Average: 32.27
Average: 33.80
>=Q30
77.73%
>=Q30
83.75%
Total (million)
Input Reads Q score
Trimmed Reads Q score
