# Supplementary material for: Rapid evaluation and quality control of next generation sequencing data with FaQCs
Source: BMC Bioinformatics. 2014 Nov 19;15(1):366. doi: 10.1186/s12859-014-0366-2 (PMC4246454; doi:10.1186/s12859-014-0366-2)
Supplement: Additional file 2: Table S1 and Table S2. — The de novo assembly and read mapping effects of FaQCs data processing. [file 12859_2014_366_MOESM2_ESM.docx]

Additional file 2 Table S1. De novo assembly on the Ion Torrent data and the metagenome dataset.

|  | # Of Contigs | N50 | Total assembly bases | Reference_Coverage % | # Of SNPs |
| --- | --- | --- | --- | --- | --- |
| *E.coli* Ion Torrent PGM | |  |  |  |  |
| Normalized_raw | 10739 | 3273 | 1906012 | 38.99% | 412 |
| Trimmed | 15057 | 3687 | 3173962 | 62.48% | 397 |
| HMP Mock illumina GAII | |  |  |  |  |
| Normalized_raw | 28690 | 3402 | 40276659 | 49.22% | 30740* |
| Trimmed | 31622 | 2569 | 40580186 | 49.63% | 27966* |

* The SNPs are listed as a reference point for the metagenome samples for two primary reasons: 1) real differences may be present between the sequenced samples and the available references; 2) due to the difference in reference coverage and because some regions of the references may differ more than others, this comparison may not perfectly reflect the influence of trimming.

Additional file 2 Table S2. Reads Mapping Result

|  | Ref | Ref len | % of reads mapped | Genome Coverage % | Avg fold(x) | # of SNPs |
| --- | --- | --- | --- | --- | --- | --- |
| ***E.coli* MiSeq (reads input: 11,041,676)** | | |  |  |  |  |
| **Normalized_raw** | Chromosome | 4639675 | 98.49% | 100.00% | 350.57 | 4 |
| **Trimmed** | Chromosome | 4639675 | 99.63% | 100.00% | 337.23 | 3 |
|  | | |  |  |  |  |
| ***P.Stuartii* MiSeq (reads input: 16,134,492)** | | |  |  |  |  |
| **Normalized_raw** | Chromosome | 4285950 | 90.17% | 100.00% | 314.73 | 1 |
|  | Plasmid | 48865 |  | 100.00% | 955.92 | 0 |
| **Trimmed** | Chromosome | 4285950 | 98.02% | 100.00% | 340.78 | 1 |
|  | Plasmid | 48865 |  | 100.00% | 1039.11 | 0 |
|  | | |  |  |  |  |
| ***E.coli* Ion Torrent PGM (reads input: 1,370,899)** | | |  |  |  |  |
| **Normalized_raw** | Chromosome | 5273097 | 85.95% | 98.76% | 6.92 | 109 |
| **Trimmed** | Chromosome | 5273097 | 93.38% | 96.56% | 4.52 | 26 |
|  | | |  |  |  |  |
| **HMP Mock illumina GAII (reads input: 135,027,77)** | | |  |  |  |  |
| **Normalized_raw** | Mock Refs | 83,859,390 | 68.13% | 72.41% | 7.44 | 13283* |
| **Trimmed** | Mock Refs | 83,859,390 | 79.23% | 72.79% | 8.03 | 13745* |

* The SNPs are listed as a reference point for the metagenome samples for two primary reasons: 1) real differences may be present between the sequenced samples and the available references; 2) due to the difference in reference coverage and because some regions of the references may differ more than others, this comparison may not perfectly reflect the influence of trimming.
