# Supplementary figures and images for: Rapid evaluation and quality control of next generation sequencing data with FaQCs
Source: BMC Bioinformatics. 2014 Nov 19;15(1):366. doi: 10.1186/s12859-014-0366-2 (PMC4246454; doi:10.1186/s12859-014-0366-2)

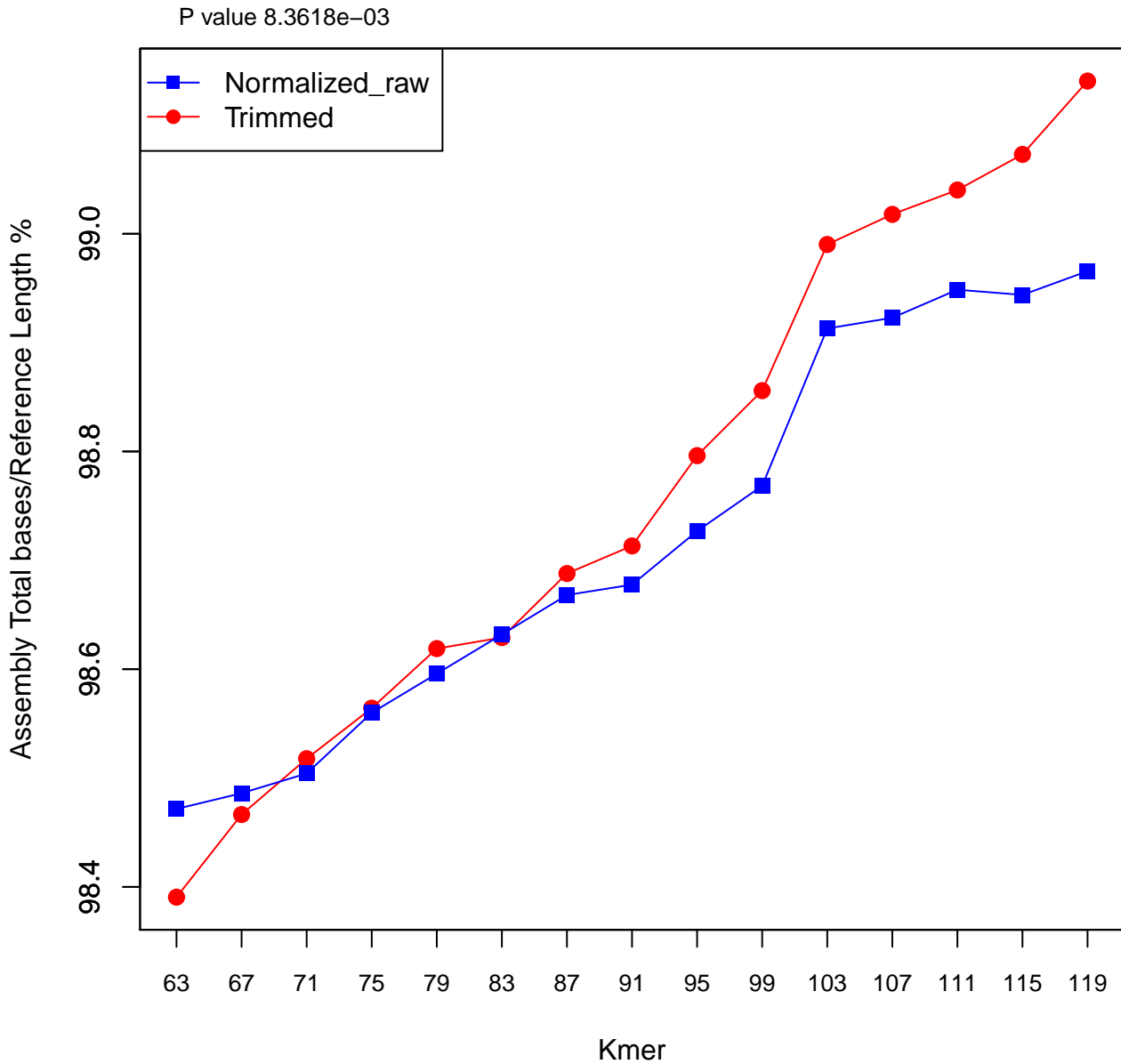

*E.coli*

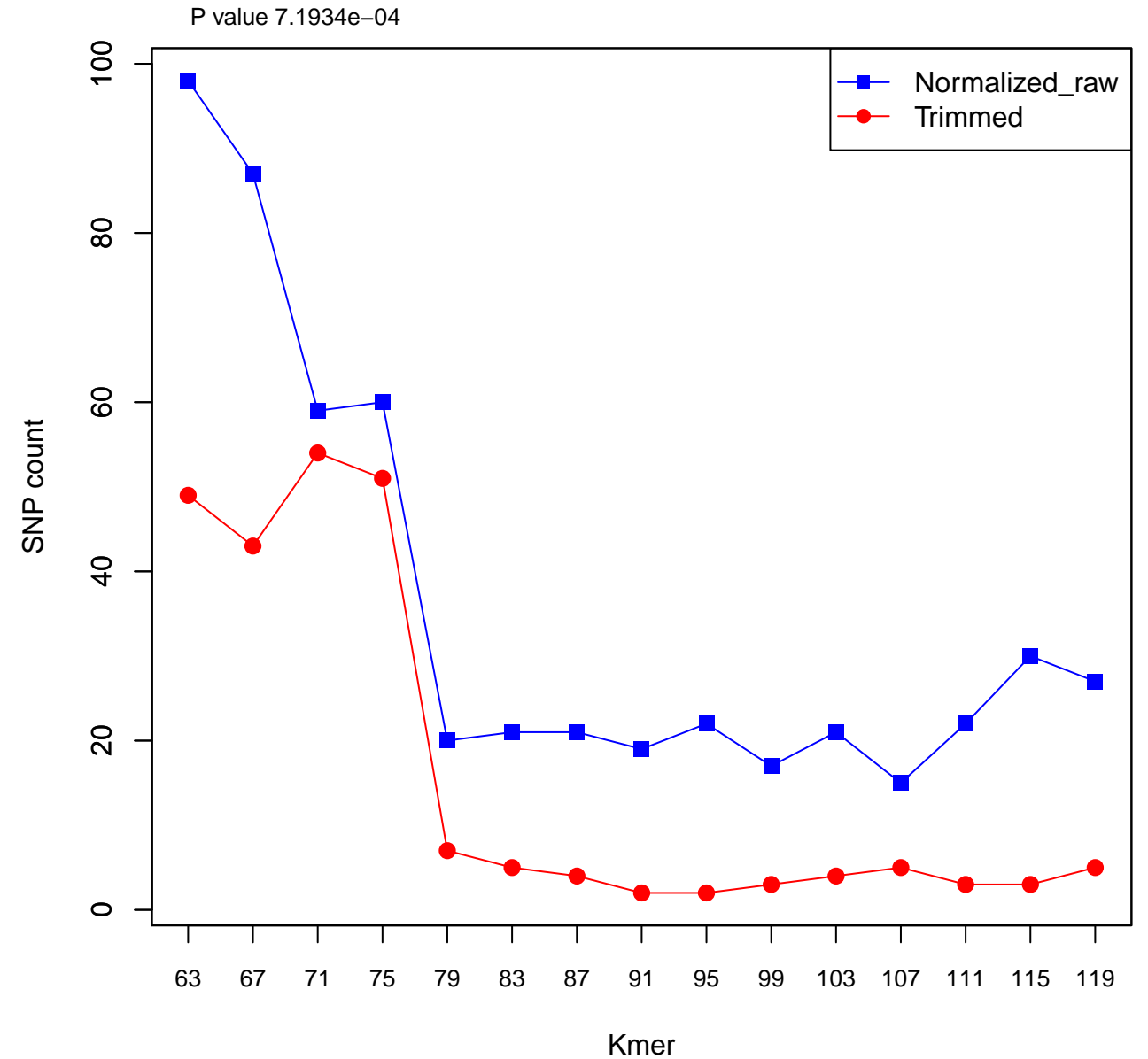

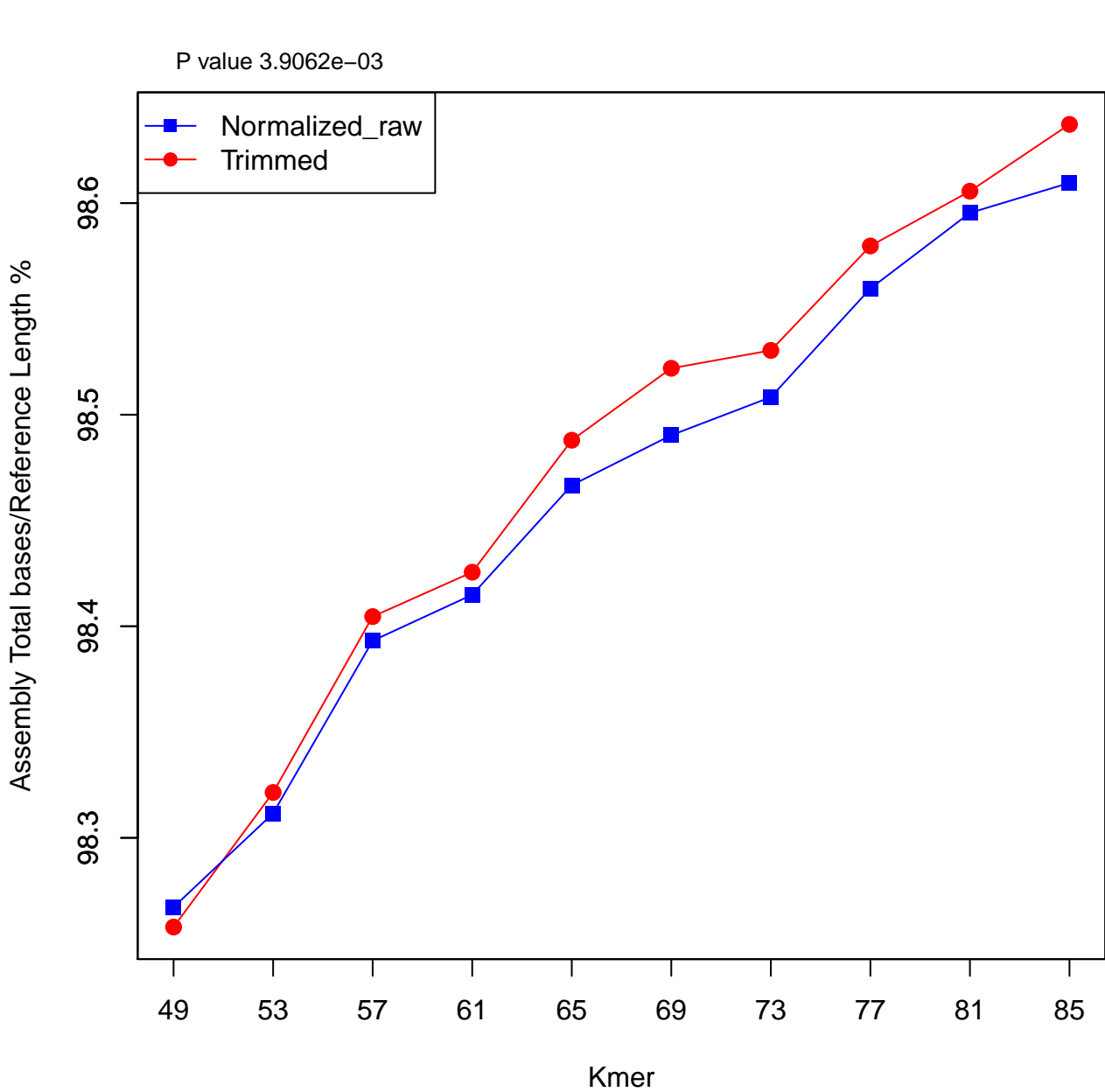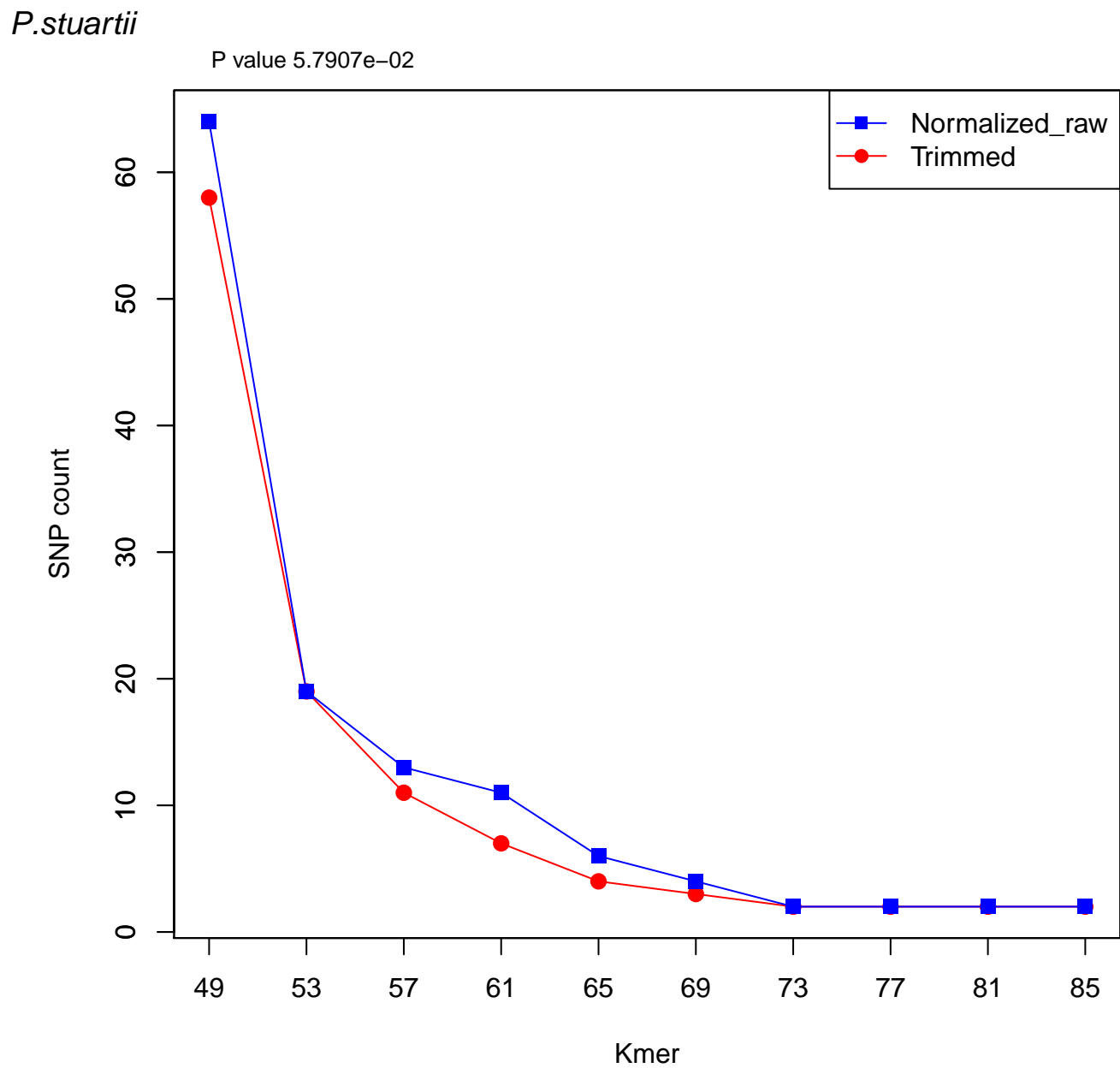

Supplement: Additional file 3: Figure S2. — Comparisons of assembly completeness and assembly SNP error before and after FaQCs data processing. [file 12859_2014_366_MOESM3_ESM.pdf]
